# Modeling the Predictive Value of Evidence-Based Referral Criteria to Support Healthy Gestational Weight Gain among an Australian Pregnancy Cohort

**DOI:** 10.3390/nu14020381

**Published:** 2022-01-17

**Authors:** Shanna Fealy, Jenna Hollis, Julia Martin, Lucy Leigh, Christopher Oldmeadow, Clare E. Collins, Roger Smith, Shelley Wilkinson, Alexis Hure

**Affiliations:** 1 School of Medicine and Public Health, College of Health and Wellbeing, University of Newcastle, Callaghan, NSW 2308, Australia; sfealy@csu.edu.au (S.F.); jenna.hollis@health.nsw.gov.au (J.H.); Julia.Martin@uon.edu.au (J.M.); roger.smith@newcastle.edu.au (R.S.); 2 School of Nursing, Paramedicine and Healthcare Sciences, Faculty of Science and Health, Charles Sturt University, 7 Major Innes Road, Port Macquarie, NSW 2444, Australia; 3 Hunter Medical Research Institute, Lot 1 Kookaburra Circuit, New Lambton Heights, NSW 2305, Australia; lucy.leigh@hmri.org.au (L.L.); christopher.oldmeadow@hmri.org.au (C.O.); clare.collins@newcastle.edu.au (C.E.C.); 4 Hunter New England Population Health, Hunter New England Local Health District, Wallsend, NSW 2287, Australia; 5 School of Health Sciences, College of Health and Wellbeing, University of Newcastle, Callaghan, NSW 2308, Australia; 6 Department of Endocrinology, John Hunter Hospital, Newcastle, NSW 2305, Australia; 7 School of Human Movement and Nutrition Sciences, Faculty of Health and Behavioural Sciences, The University of Queensland, St. Lucia, QLD 4067, Australia; s.wilkinson@uq.edu.au

**Keywords:** gestational weight gain, pregnancy, antenatal care, maternal nutrition, referral, dietitian

## Abstract

Globally, there has been a renewed focus on addressing gestational weight gain (GWG). In Australia, the Department of Health pregnancy care guidelines recommend women be offered routine weighing and receive brief nutritional and physical activity support during antenatal care visits. Women gaining weight outside the Institute of Medicine (IOM)’s weight gain reference values are further recommended to be referred to a dietitian. However, professional and organizational barriers, including an absence of weight gain referral pathways and limited workforce resources, exist with the translation and scaling of these recommendations into practice. This study aimed to explore patterns of GWG among a cohort of Australian pregnant women and to determine if pregnancy weight gains of above or below 2 kg or 5 kg in the second and third trimester can be used to predict total GWG outside recommendations. Sensitivity, specificity, negative, and positive likelihood ratios were calculated. The most predictive time point was 24 weeks’ gestation using the minimum weight change parameter of +/−2 kg, demonstrating reasonable sensitivity (0.81, 95% CI 0.61–0.83) and specificity (0.72, 95% CI 0.61–0.83), resulting in 55% (*n* = 72/131) of the cohort qualifying for dietetic referral. Given the current health service constraints, a review of dietetic services within maternity care is warranted.

## 1. Introduction

In 2009, the Institute of Medicine (IOM) published revised guidelines for gestational weight gain (GWG) in response to the increasing prevalence of overweight and obesity within the United States (US) population [1]. These guidelines use the World Health Organization’s (WHO) body mass index (BMI) categories, although recommendations for obesity grade (grades 1–3) are not included [1]. Weight gains above or below the IOM guidelines are associated with an increased risk of adverse maternal and infant outcomes. A 2017 systematic review and meta-analysis, including over 1.3 million women, found GWG’s above the IOM guidelines was associated with an increased risk of large for gestational age infants (odds ratio (OR) 1.85, 95% confidence interval (CI) 1.76–1.95), macrosomia (OR 1.95, 95% CI 1.79–2.11), and birth by caesarean (OR 1.30, 95% CI 1.25–1.35). Low GWG was associated with small for gestational age infants (OR 1.53, 95% CI 1.44–1.64) and preterm birth (OR 1.70, 95% CI 1.32–2.20) [2]. Emerging research including the Developmental Origins of Health and Disease (DOHaD) hypothesis and infant gut microbiome suggest that weight gains above and below the guidelines increases the risk of non-communicable diseases across the lifespan including cardiovascular disease and diabetes [3,4].

Despite the health risks, pregnant women continue to gain weight outside of recommendations. Santos et al. analyzed individual data from over 218,000 pregnancies across 33 cohorts worldwide to describe GWG patterns and develop weight gain charts for all BMI categories [5]. More than two-thirds of participants gained weight outside the IOM guidelines, with 23% gaining weight below and 44% gaining above the recommendations, referred to as excessive GWG (EGWG) [5]. Similar results have been reported, including in non-Indigenous Australian populations [2,6,7,8]. The results of one study among a cohort of Indigenous Australians by Schumacher et al. found that 32% of pregnant women exhibited low GWG with 54% gaining weight above the IOM guidelines [9]. Of concern, a recent retrospective audit of a large (*n* = 1034) multiethnic Australian cohort of pregnant women with a diagnosis of gestational diabetes mellitus (GDM), conducted by Barnes et al., identified that 61% of the women in this cohort evidenced high rates of early EGWG (mean gestational age 23.0 weeks SD 5.5) with only 44% achieving their GWG targets within the IOM recommendations by the time they gave birth [10].

Pregnancy is often regarded as an opportune time to provide dietary and physical activity behavioral support and education to benefit women, their offspring, and families [11]. This is often attributed to frequent contact with health professionals throughout the antenatal period and an increased motivation by women and their families to make positive health behavior changes for the health of their babies [12,13,14]. In many countries, including the US [1], Canada [15], and Australia [16], clinical practice guidelines recommend addressing weight gain in pregnancy through the implementation of routine weight monitoring in combination with nutrition and physical activity advice. The most recent Australian Clinical Practice guidelines for Pregnancy Care updated in 2021, recommend that antenatal care providers assess weight to detect low or high GWG at all antenatal visits; provide brief behavioral support on GWG, healthy eating, physical activity, and for women gaining weight at a rate below or above recommendations; offer a referral for dietary support from an accredited practicing dietitian (APD) [16].

To date in Australia, the translation of the GWG guideline recommendations into maternity care services has been suboptimal and ad hoc [11,17]. Women consistently identify GWG as an important and acceptable topic to be addressed during routine antenatal care through open, respectful conversations [11,18,19]. However, individual and institutional barriers impede the provision of GWG support by health professionals, such as low health provider confidence and competence in addressing GWG, lack of resources, appointment time constraints, inadequate knowledge of guidelines, and a lack of availability or knowledge of specialist support and lack of appropriate referral services (i.e., APDs), for women who require additional support [17,18,20,21,22]. In contrast, when specialist health services are actively offered and integrated into pregnancy care, women are reported to be more likely to gain weight within the IOM weight gain recommendations [18,19].

An Australian longitudinal study by Wilkinson et al. demonstrated within one large tertiary hospital that increases in professional and organizational service capacity improved dietetic support and uptake over time [19]. Increases in nutritional services, such as the integration of dietetic services into antenatal care and increased awareness of nutritional services among staff and women, improved alignment with GWG guidelines [19]. However, not all women who requested and may have benefited from nutrition support were able to be accommodated due to the resource limitations. Therefore, the authors concluded further improvements were necessary, including targeted referral pathways, to ensure that service demands can be met by professional and organizational capacity [19].

Dietitians are trained and qualified to provide individualized medical nutrition therapy and behavior change counselling and support to pregnant women in order to optimize food and nutrient intakes and support appropriate GWG. However, in Australia antenatal services are rarely (private services) or insufficiently (public services) staffed with dietitians impacting the capacity to receive referrals for pregnant women and/or limiting the time to provide consults to pregnant women gaining weight outside of GWG recommendations [17]. Additionally, there is a paucity of weight gain referral pathways to identify women at risk of gaining weight outside IOM GWG reference values throughout pregnancy to guide referral practices [17]. Gaining a better understanding of GWG trajectory patterns that predict low or high GWG by the end of pregnancy could enable early identification of women at risk for adverse GWG outcomes with subsequent referral to a dietitian for more intensive, medical nutrition therapy and allow for professional and organizational planning and resource prioritization. The aim of the current study was to explore weight gain patterns in a longitudinal cohort of pregnant Australian women and determine if pregnancy weight gains of above or below 2 kg or 5 kg in the second and third trimester can be used to predict total GWG within or outside of the IOM recommendations.

## 2. Materials and Methods

This is a secondary analysis of data obtained from the Women and Their Children’s Health (WATCH) study. The relevant WATCH study population and data collection methods used for the current analysis are outlined as follows.

### 2.1. Study Population

The WATCH study was a small (*n* = 180 women and *n* = 182 children) but detailed Australian prospective longitudinal cohort study [23]. The study was conducted to investigate the DOHaD hypothesis, specifically maternal and child health behavior and nutrition trends [23]. Women in the study were recruited to participate during early pregnancy (<18 weeks) with follow up of participants occurring up to 4 years post birth. The detailed WATCH study protocol has previously been published [23]. Briefly, women were primarily recruited from one large tertiary hospital antenatal clinic in New South Wales, Australia, between June 2006 and December 2007. A small number of women were recruited via word of mouth and local media coverage between these dates. Pregnancy data were collected during study visits occurring at approximately 19, 24, 30, and 36 weeks’ gestation with birth and postnatal data collected at approximately 3, 6, 9, and 12 months and 2, 3, and 4 years [23]. The research protocol for the WATCH study was approved by the Hunter New England Human Research Ethics Committee (approval number: 06/05/24/5.06). All participants provided written informed consent.

### 2.2. Anthropometric Measures

Maternal anthropometrics were obtained by physical assessment during all study visits by a team of APDs with Level One Anthropometrics certification from the International Society for the Advancement of Kinanthropometry (ISAK) [23]. Pre-pregnancy weight was self-reported by participants and documented at the first pregnancy visit with all other weight measurements obtained at study visits by researchers using the same set of annually calibrated electronic scales as follows: Visit 1 (19 weeks’ gestation), Visit 2 (24 weeks gestation), Visit 3 (30 weeks’ gestation), and Visit 4 (36 weeks’ gestation) [23]. Maternal height and weight were taken in clothing with no shoes. Height was measured on two consecutive appointments to the nearest 1 mm with an average of the two measures used. Where both height measures varied more than 1.5%, a third measure was taken, and the median used as the maternal height reference [23]. Pre-pregnancy BMI was calculated using the self-reported pre-pregnancy weight and the practitioner measured maternal height reference [23]. The population sample for the current analysis consisted of 131 women who recorded a self-reported pre-pregnancy weight, total pregnancy weight at 36 weeks’ gestation (Visit 4) and recorded at least one measure of weight at any of the WATCH study pregnancy visits (Visits 1–3).

For this analysis, estimations of expected “appropriate” GWG were modeled from the IOM weight gain in pregnancy guidelines [1]. The IOM guidelines recommend that women with a pre-pregnancy BMI in the underweight category (BMI < 18.5 kg/m^2^) aim for a total GWG of between 12.5 kg and 18 kg, normal weight category (BMI 18.5 – < 25 kg/m^2^) gain between 11.5 kg and 16 kg, overweight category (BMI 25 – < 30 kg/m^2^) between 7 kg and 11.5 kg, and obese category (BMI ≥ 30 kg/m^2^) between 5 kg and 9 kg [1]. The minimum and maximum weekly rates of GWG were calculated for each week of gestation from the second trimester of pregnancy onwards (13–40 weeks’ gestation) according to pre-pregnancy BMI category using Microsoft excel as per Supplementary Materials File S1. The GWG of WATCH participants was then calculated and compared to the expected “appropriate” range, according to pre-pregnancy BMI and weeks of gestation.

### 2.3. Weight Gain Parameters

Weight gain cut-off points of 2 kg and 5 kg above or below the minimum or maximum weekly GWG targets at Visits 1–3 and accuracy for predicting over- or underweight (+/−2 kg or +/−5 kg) at 36 weeks’ gestation (Visit 4) were the primary outcome variables utilized. These parameters were initially derived from clinical discussion and expert dietetic opinion. A decision prompt algorithm for resource sharing and/or dietitian referral was developed in one large Australian tertiary maternity hospital using the hospital’s electronic medical record embedded weight tracker. These electronic records used +/−2 kg or +/−5 kg across a defined number of visits and recorded pre-pregnancy BMI categories. The cut-offs were adopted for this study to test this practice-informed GWG criteria.

### 2.4. Statistical Analysis

The primary statistical objectives were to determine which WATCH study visit (Visits 1–3) and corresponding gestation was best for predicting over- or underweight at 36 week’s gestation (Visit 4). If a participant’s GWG was greater than 2 kg or 5 kg below the minimum, or if they were 2 kg or 5 kg above the maximum expected range at Visits 1, 2, or 3, then they would score a positive (1) test value. If weight was within the expected range, then they would score a negative (0) test value. Sensitivity (proportion of participants exhibiting GWG +/−2 kg or +/−5 kg across time points 1–3 with a positive result (+/−2 kg or +/−5 kg) at time point 4) and specificity (proportion of participants exhibiting GWG within a normal range across time points 1–3 with a normal GWG at time point 4) were calculated. Negative and positive likelihood ratios were additionally calculated to assist in determining test accuracy. Positive likelihood ratios were used to predict how much more likely it was for a positive test across time points (1–3) among participants who exhibit weight changes of +/−2 kg or +/−5 kg than it was for those who gained weight within the normal ranges. Negative likelihood ratios were used to predict the likelihood of a negative test result amongst participants who exhibit weight changes of +/−2 kg or +/−5 kg across time points (1–3). The sensitivity, specificity, positive, and negative likelihood ratios were also calculated to determine the distribution of positive tests based on two consecutive time points for +/−2 kg outside the expected range at Visits 1 and 2 and Visits 2 and 3, respectively, for predicting Visit 4. Sensitivity, specificity, positive and negative likelihood ratios, and 95% CIs were estimated using the PROC NLMIXED on SAS V9.4.

## 3. Results

The distribution of GWG (above and below the maximum and minimum) at each study visit is displayed in Table 1. Proportionally, the patterns of GWG indicated that women with a pre-pregnancy BMI ≥ 30 kg/m^2^ exhibited weight gains below 2 kg and 5 kg across all WATCH study visits. In contrast, women with a pre-pregnancy BMI < 18.5 kg/m^2^ demonstrated weight gains above 2 kg and 5 kg across all study visits. Women with a pre-pregnancy BMI in the normal weight category (BMI 18.5 – < 25 kg/m^2^), were most likely to exhibit weight gains within the IOM GWG reference ranges.

Sensitivity, specificity, positive and negative likelihood ratio estimates at WATCH study Visits 1–3, and GWG at Visit 4 (i.e., 36 weeks’ gestation) are presented in Table 2. For the +/−2 kg and +/−5 kg models, sensitivity and specificity estimates increased across all time points. For the +/−2 kg cut-off point across WATCH study Visits 1–3, positive likelihood ratios increased while negative likelihood ratios decreased, as expected. Specificity for detecting +/−2 kg at WATCH Visit 1 (i.e., 19 weeks’ gestation), was observed to be particularly low (0.59, 95% CI 0.47–0.71), indicating that a positive test at 19 weeks would lead to the unnecessary referral of women who did not end up gaining weight outside the recommended range. For the +/−5 kg cut-off points, sensitivity at 19 and 24 weeks were also observed to be low (Visit 1, 0.41, 95% CI 0.25–0.56; Visit 2, 0.65, 95% CI 0.49–0.80), indicating that using a +/−5 kg margin at 19 and 24 weeks were poor at predicting high or low GWGs at 36 weeks’ gestation. In contrast, the specificity using a +/−5 kg margin at 19 and 24 weeks were high at 0.91, 95% CI 0.86–0.97 and 0.94, 95% CI 0.89–0.99, respectively, indicating that a negative test at these time points are better for detecting GWG within the reference ranges.

Estimates for time point 3 (i.e., 30 weeks’ gestation) for both the +/−2 kg and +/−5 kg models both exhibited relatively high sensitivity and specificity. Positive likelihood ratios at the 30 week time points with the +/−2 kg cut-off indicated that those with a positive test result were 5.83 times more likely to occur in those exhibiting high or low GWG than those without with the +/−5 kg cut-off indicating that a positive test result at this time point was 27.1 times more likely to occur in those exhibiting total GWG outside the recommendations than those gaining within the normal weight gain range. Negative likelihood ratios at the 30 week time point indicate that the normal GWG is 0.11 times (+/−2 kg) and 0.14 times (+/−5 kg) less likely to occur in those that exhibit GWG outside the recommendations. The sensitivity (0.81, 95% CI 0.71–0.90) and specificity (0.72, 95% CI 0.61–0.83) for a +/−2 kg cut-off at 24 weeks gestation were high (sensitivity, 0.81, 95% CI 0.71–0.90; specificity, 0.72, 95% CI 0.61–0.83) with a positive likelihood ratio of 2.87, 95% CI 1.69–4.04 and negative likelihood ratio 0.27, 95% CI 0.13–0.41. Cross tabulation’s describing the distribution between GWG at Visits 1, 2, and 3 and final visit (upon which the sensitivity, specificity, and likelihood ratios were based) are reported in Supplementary Materials File S2.

Table 3 displays the distribution of positive tests based on two consecutive time points for Visits 1 and 2 and Visits 2 and 3, respectively. Using a +/−2 kg weight gain at Visit 1 and 2 as the indicator for referral, 65 women would have met the criteria for referral (as opposed to 77 using just using Visit 1 or 72 using just Visit 2). Using a +/−2 kg weight gain at Visits 2 and 3 as the indicator for referral, 59 women would have met the criteria for referral (as opposed to 72 using just Visit 2 or 71 using just Visit 3).

The sensitivity, specificity, and positive and negative likelihood ratios for two consecutive visits outside the expected range with a +/−2 kg cut off are reported in Table 4. In both cases, using the consecutive visits gave a smaller point estimate for sensitivity compared to either of the individual visits, but a higher point estimate for the specificity. This means that using data from two consecutive visits would reduce the number of referrals given to women unnecessarily but would also miss more women who might benefit from a referral. Cross tabulations describing the distribution between GWG at Visits 1, 2, and 3 and the final visit upon which these sensitivity, specificity, and likelihood ratios were based are included in Supplementary Materials File S2.

## 4. Discussion

The primary aim of the current study was to model and evaluate whether GWG above or below 2 kg or 5 kg, identified during antenatal care visits (19, 24, or 30 weeks) could be used to predict total GWG (measured at 36 weeks’ gestation) outside the IOM recommendations to better inform GWG guideline translation, particularly dietetic referral pathway development. The current analysis identified that the earliest single time point most predictive of total GWG outside the IOM reference ranges was 24 weeks’ gestation using the minimum weight change variable of +/−2 kg, demonstrating reasonable sensitivity (0.81, 95% CI 0.61–0.83) and specificity (0.72, 95% CI 0.61–0.83). The positive likelihood ratio indicated that women with GWG 2 kg outside the recommendations at 24 weeks were 2.87 times more likely to gain weight above and below GWG targets than those who gained weight within the guidelines. The negative likelihood ratio indicates that women who gained weight within the guidelines were only 0.27 times more likely to gain weight outside the IOM reference values at 36 weeks’ gestation. However, given the current lack of APD services currently identified, particularly in the provision of maternity dietetic services in Australia [17], using one time point alone is not likely to be feasible and suggests that a review of APD services within maternity care is warranted. Exclusive use of the 24 week time point alone would mean that 55% (*n* = 72/131) of the WATCH cohort would qualify for dietetic referral. Arguably, these numbers would require significant professional and organizational planning and prioritization of resources to meet referral demand. Importantly, with investment in APD services within maternity care using the +/−2 kg time point alone, it would provide the opportunity to not only optimize GWG but also improve glycemic profiles of women diagnosed with GDM [10].

To address the current limitations identified with maternal dietetic service delivery, a stepped implementation approach to dietetic referral (i.e., ensuring that women most at risk of high and low GWGs receive dietetic referral while allowing for concurrent service upscaling) is warranted. During initial service upscaling, it may be pragmatic in the short term to base referrals off two consecutive pregnancy time points. For example, using the +/−2 kg weight change parameter at 19 and 24 weeks’ gestation would reduce the referral rate proportionally by approximately 5% with 65 women or 49.6% of the cohort qualifying for referral. A further reduction in referrals of up to 10% is evidenced when using the +/−2 kg weight change parameter at 24 and 30 weeks’ gestation time points, reducing the referral rate to 59 women. While the sensitivity and specificity for use of two consecutive time points are reasonable, there will be women in need of specialist care from APDs who will have missed an opportunity to improve their health and the health of their infants. Recent data from the Australian Institute of Health and Welfare (AIHW), Mothers and Babies 2021 report indicates that obtaining maternal weight to inform dietetic referral at two consecutive second trimester time points is feasible, with 76.6% of Australian women first accessing antenatal care before 14 weeks’ gestation and over 90% of women first accessing antenatal care between 14–19 weeks’ gestation [24]. Referral after 24 weeks’ gestation rather than after 30 weeks’ gestation also may afford more time for behavioral change to occur and may also assist with detection and/or management of women at concurrent risk of associated adverse pregnancy outcomes such as GDM [10].

A recent retrospective cohort study conducted by Barnes et al. investigated patterns of GWG pre- and post-diagnosis of GDM and other outcome variables including interprofessional GDM management, insulin therapy, and large for gestational age infants (LGA), among a multiethnic cohort of Australian pregnant women (*n* = 1034) [10]. In this study, personalized weekly GWG targets were calculated for women according to the IOM guidelines using their self-reported pre-pregnancy BMI. The findings indicated that patterns of early GWG, above a women’s personalized GWG target prior to GDM diagnosis (mean gestation of diagnosis = 23 weeks), were more likely to occur among women with a mean pre-pregnancy BMI in the overweight category (mean BMI = 27.8 kg/m^2^). Overweight women were also more likely to exhibit high total GWG (mean = 18.3 kg, last measured within 4 weeks from birth), compared to those that gained within or below their total personalized GWG targets [10]. Women who were observed to gain in excess of their weight gain targets (prior to giving birth) and who received interprofessional GDM management post-GDM diagnosis, were found to have a higher incidence of having a large for gestational age infant when compared to women who received interprofessional GDM management but gained below or within their personalized weight gain targets (adjusted odds ratio 1.99, 95% CI 1.25–3.15, *p* = 0.004) [10]. Based on these findings the authors discussed that while GWG management post-diagnosis of GDM (typically occurring between 24–28 weeks) is effective at slowing rates of GWG, it may be more beneficial to address GWG prior to GDM diagnosis [10]. Moreover, the authors suggest that the earlier women at risk of high GWG can be identified, allows for prompt referral for dietetic support and dietary behavioral change to occur. In particular, there is an opportunity for women who are identified as at risk for high GWG early in pregnancy, who are subsequently diagnosed with GDM at mid to late pregnancy, to receive early dietary behavioral support to achieve appropriate weight gain targets and reduce their risk of having an LGA infant [10].

Although further research is required to test the referral process, our model suggests that there is potential for weight gains of 2 kg or greater identified at two consecutive second trimester time points occurring at approximately 19 and 24 weeks’ gestation to be used to identify women at risk for high total GWG and risk of GDM. This supports the Academy of Nutrition Dietetics GDM evidenced practice-based guidelines by increasing the scope of recommendations to encompass screening and detection of at risk women prior to GDM diagnosis for early glycemic control and GWG management [25]. The detection of women at risk of high GWG by or close to 24 weeks gestation, allows for early dietetic assessment (i.e., before GDM diagnosis), the setting of personalized nutritional goals (such as related to optimizing intakes and/or dietary patterns modification and physical activity planning) and ongoing behavior change counselling support and monitoring of these goals throughout the remainder of pregnancy [25]. Early screening additionally may support women to plan around their competing psychosocial circumstances, such as employment, pregnancy symptoms, transport, financial, and family circumstances, that are known barriers to meeting GWG targets and dietetic follow-up regimes [25,26]. There is similarly opportunity to support women at risk of low GWG to achieve weight gain targets and prevent small for gestational age infants.

Although there is limited time during the third trimester (after 30 weeks’ gestation) for providing more than brief dietetic assessment and follow up, there is potential scope for the identification of late pregnancy weight gains above the IOM recommendations to be used for identifying women at risk of adverse birth outcomes as well as post-partum weight retention. A previous analysis of the WATCH cohort conducted by Martin et al. identified that only 22% of women followed up at 6 months post-partum (*n* = 29) had returned to or weighed less than their pre-pregnancy BMI [27]. Only 32% (*n* = 37) returned to or weighed less than their pre-pregnancy BMI at 12 months post-partum, with GWG significantly predicting weight retention at 12 months [27]. Given these results, there is further opportunity to use the +/−2 kg and +/−5 kg weight change parameters during late pregnancy (perhaps at 30 and 36 weeks) and evaluate their predictive value for post-partum weight retention and infant birthweight. This may be particularly useful for women who have had a GDM diagnosis or birthed a large or small for gestational age infant to receive ongoing weight gain and dietary support to optimize maternal and infant health for subsequent pregnancies and reduce the risk of non-communicable disease development, such as type 2 diabetes, in later life. Statistically, both the 2 kg and 5 kg weight parameters measured at 30 weeks’ gestation performed particularly well in the current analysis, most obviously being closer to 36 weeks’ gestation, where total GWG was assessed; thus, further research is required to confirm these hypotheses.

There is currently a lack of clear guidance on how to address GWG at the service provider level using interprofessional models of care, with wide variations in service delivery models and service capacity evident within Australia [17]. In Australia, antenatal care (including referral to dietitians) can be accessed through both the public health system (a universal health care system, federally funded through the Medicare program) and the private health system (funded personally or through individual private health insurance) [28]. Antenatal care generally consists of between 7 and 10 visits for low-risk women across pregnancy. Midwives and obstetricians are typically the main maternity care providers for pregnant women either independently or within shared care models (i.e., between general practitioners (GPs) and Aboriginal Health Workers or Midwives or Obstetricians) [16], who can refer pregnant women to specialist services such as dietitians if needed. The literature indicates that these health professionals, including dietitians, have identified a lack of public health service resourcing and time pressures as barriers to antenatal screening, education, and care provision [29,30]. A lower prioritization of weight management support during pregnancy as a referring health issue and a lack of public dietetic service capacity in many areas of Australia prevents timely dietetic consultation [17]. The need for a more appropriately resourced maternity care service to address GWG has been consistently identified particularly with the communication of public health messaging around GWG and guideline adherence [11,31]. There is also an urgent need for the development of Australian specific referral pathways for maternal health to be developed [17], particularly given the most recent Australian pregnancy care guidelines recommend that women who exhibit GWGs outside the IOM guidelines to be referred to an APD [16]. The data presented in this analysis may assist with organizational GWG service planning and the development of referral pathways for consideration in the development of Australian-specific GWG guidelines that support maternal and infant health.

### 4.1. Strengths

To our knowledge this is the first study to model weight gain parameters as referral points predictive of total GWG outside the IOM weight gain in pregnancy guidelines. The study has used a combination of self-reported (pre-pregnancy) and objectively measured weights obtained during WATCH study pregnancy care visits.

### 4.2. Limitations

Total GWG was measured at 36 weeks’ gestation and as such, may not accurately reflect total pregnancy weight gain immediately prior to giving birth. We acknowledge that the use of self-reported pre-pregnancy weight to inform pre-pregnancy BMI and weight gain targets as limitations; however, a systematic review by Headen et al. [32] concluded that while women were more likely to underestimate their pre-pregnancy weight, the magnitude of error between self-reported and measured weights was assessed as minimal (−2.94–0.29 kg) with low risk of bias for GWG and birth outcomes. Self-reported pre-pregnancy weight is a practical and cost-effective research measurement [32]. In addition, the study was conducted in one small Australian cohort from one hospital and as such, the results may not be generalizable to other more culturally diverse cohorts of women. In particular, the data used for women with a pre–pregnancy BMI < 18.5 kg/m^2^ was derived from a sample of *n* = 7 women and should be interpreted with caution. Further research is now required within larger multiethnic cohorts of pregnant women.

## 5. Conclusions

Incongruencies exist with the translation of GWG guideline recommendations into maternity care, particularly at the service provider level. A deficit of screening and referral pathways to identify women most in need of dietetic support to achieve appropriate GWG is evident. The current analysis suggests that weight changes of +/−2 kg detected at one second trimester time point or across two second trimester time points may be useful to inform dietetic referral practices and promote GWG guideline adherence. Given the professional and organizational barriers that exist with dietetic resourcing within antenatal care, a stepped approach for dietetic referral is warranted, allowing for service providers to prioritize resources to meet demand. The implementation of referral pathways early in the second trimester may additionally allow “at-risk” women to receive early dietary behavioral support to achieve appropriate weight gain targets and glycemic control, potentially reducing their risk of having a large or small for gestational age infant and optimizing overall maternal and infant health. Further research is now required to test the predictive value of the weight change referral parameters within larger multi-ethnic cohorts of pregnant women.

## Figures and Tables

**Table 1:** Distribution of gestational weight gain by WATCH study visit.

WATCH Study Visit (Week’s Gestation) +/− 2 kg or 5 kg		Pre-Pregnancy BMI <18.5 (*n* = 7)	Pre-Pregnancy BMI 18.5–<25 (*n* = 68)	Pre-Pregnancy BMI 25–<30 (*n* = 29)	Pre-Pregnancy BMI ≥30 (*n* = 27)
Visit 1 (19 weeks) +/−2 kg	>2 kg below the minimum	0	12 (18%)	4 (14%)	11 (41%)
	Within limits	1 (14%)	34 (50%)	9 (31%)	10 (37%)
	>2 kg above maximum	6 (86%)	22 (32%)	16 (55%)	6 (22%)
Visit 2 (24 weeks) +/−2 kg	>2 kg below the minimum	0	8 (12%)	3 (10%)	8 (30%)
	Within limits	1 (14%)	37 (54%)	10 (34%)	11 (41%)
	>2 kg above maximum	6 (86%)	23 (34%)	16 (55%)	8 (30%)
Visit 3 (30 weeks) +/−2 kg	>2 kg below the minimum	0	7 (10%)	1 (3.4%)	7 (26%)
	Within limits	1 (14%)	36 (53%)	13 (45%)	10 (37%)
	>2 kg above maximum	6 (86%)	25 (37%)	15 (52%)	10 (37%)
Visit 4 (36 weeks) +/−2 kg	>2 kg below the minimum	0	6 (8.8%)	3 (10%)	6 (22%)
	Within limits	2 (29%)	40 (59%)	11 (38%)	11 (41%)
	>2 kg above maximum	5 (71%)	22 (32%)	15 (52%)	10 (37%)
Visit 1 (19 weeks) +/−5 kg	>5 kg below the minimum	0	0	1 (3.4%)	4 (15%)
	Within limits	4 (57%)	59 (87%)	25 (86%)	20 (74%)
	>5 kg above maximum	3 (43%)	9 (13%)	3 (10%)	3 (11%)
Visit 2 (24 weeks) +/−5 kg	>5 kg below the minimum	0	0	0	5 (19%)
	Within limits	4 (57%)	55 (81%)	24 (83%)	18 (67%)
	>5 kg above maximum	3 (43%)	13 (19%)	5 (17%)	4 (15%)
Visit 3 (30 weeks) +/−5 kg	>5 kg below the minimum	0	0	0	3 (11%)
	Within limits	4 (57%)	55 (81%)	20 (69%)	17 (63%)
	>5 kg above maximum	3 (43%)	13 (19%)	9 (31%)	7 (26%)
Visit 4 (36 weeks) +/−5 kg	>5 kg below the minimum	0	0	0	3 (11%)
	Within limits	4 (57%)	54 (79%)	19 (66%)	17 (63%)
	>5 kg above maximum	3 (43%)	14 (21%)	10 (34%)	7 (26%)

**Table 2:** Sensitivity, specificity, positive, and negative likelihood ratios for predicting total GWG 36 weeks’ gestation.

Outcome Variable	Model	Measure	Estimate (95% CI)
Outcome was +/−2 kg at Visit 4	Referral at 19 weeks (+/−2 kg)	Sensitivity	0.76 (0.66, 0.86)
		Specificity	0.59 (0.47, 0.71)
		LR+	1.87 (1.26, 2.48)
		LR−	0.40 (0.21, 0.59)
	Referral at 24 weeks (+/−2 kg)	Sensitivity	0.81 (0.71, 0.90)
		Specificity	0.72 (0.61, 0.83)
		LR+	2.87 (1.69, 4.04)
		LR−	0.27 (0.13, 0.41)
	Referral at 30 weeks (+/−2 kg)	Sensitivity	0.91 (0.84, 0.98)
		Specificity	0.84 (0.75, 0.93)
		LR+	5.83 (2.48, 9.17)
		LR−	0.11 (0.02, 0.19)
Outcome is +/−5 kg at Visit 4	Referral at 19 weeks (+/−5 kg)	Sensitivity	0.41 (0.25, 0.56)
		Specificity	0.91 (0.86, 0.97)
		LR+	4.76 (1.10, 8.43)
		LR−	0.65 (0.47, 0.83)
	Referral at 24 weeks (+/−5 kg)	Sensitivity	0.65 (0.49, 0.80)
		Specificity	0.94 (0.89, 0.99)
		LR+	10.2 (1.93,18.39)
		LR−	0.38 (0.21, 0.54)
	Referral at 30 weeks (+/−5 kg)	Sensitivity	0.86 (0.75, 0.98)
		Specificity	0.97 (0.93, 1.00)
		LR+	27.1 (0.00,57.47)
		LR−	0.14 (0.03, 0.25)

LR+ = positive likelihood ratio; LR− = negative likelihood ratio.

**Table 3:** Referrals based on two consecutive time points (Visit 1 and Visit 2; Visit 2 and Visit 3).

**Referral at Visit 1** **(19 Weeks)**	**Referral at Visit 2** **(24 Weeks)**	**Referral Based on Visits 1 and 2**	**Frequency (%)**
No referral	No referral	0	47 (35.88)
No referral	Refer	0	7 (5.34)
Refer	No referral	0	12 (9.16)
Refer	Refer	1	65 (49.62)
**Referral at Visit 2** **(24 weeks)**	**Referral at Visit 3** **(30 weeks)**	**Referral Based on Visits 2 and 3**	**Frequency (%)**
No referral	No referral	0	47 (35.88)
No referral	Refer	0	12 (9.16)
Refer	No referral	0	13 (9.92)
Refer	Refer	1	59 (45.04)

**Table 4:** Sensitivity, specificity, and positive and negative likelihood ratios for referral at two consecutive time points (+/−2 kg only) predicting GWG above or below (+/−2 kg) the recommended range at 36 weeks’ gestation.

Model	Measure	Estimate (95% CI)
Referral at 19 and 24 weeks (+/−2 kg)	Sensitivity	0.73 (0.63, 0.84)
	Specificity	0.75 (0.64, 0.86)
	LR+	2.93 (1.61, 4.24)
	LR−	0.36 (0.21, 0.51)
Referral at 24 and 30 weeks (+/−2 kg)	Sensitivity	0.78 (0.68, 0.88)
	Specificity	0.89 (0.81, 0.97)
	LR+	7.10 (2.05,12.14)
	LR−	0.25 (0.14, 0.37)

LR+ = positive likelihood ratio; LR− = negative likelihood ratio.

## Supplementary Materials

The following are available online at https://www.mdpi.com/article/10.3390/nu14020381/s1, Supplementary File S1: Range for expected healthy weight gain at 13–40 weeks’ gestation (and cut-points based on +/−2 and 5 kg) for all pre-pregnancy BMI categories; Supplementary File S2: Cross tabulations describing the distribution of weight gain at Visits 1, 2, and 3 versus weight gain at Visit 4.
