# Supplementary material for: Modeling the Predictive Value of Evidence-Based Referral Criteria to Support Healthy Gestational Weight Gain among an Australian Pregnancy Cohort
Source: Nutrients. 2022 Jan 17;14(2):381. doi: 10.3390/nu14020381 (PMC8779448; doi:10.3390/nu14020381)
Supplement: Supplementary file 1 [file nutrients-14-00381-s001.zip › nutrients-1534608-supplementary.pdf]

Supplementary File S1: Range for expected healthy weight gain at 13-40 weeks gestation (and cut-points based on +/- 2 and 5 kg) for all Pre-pregnancy BMI categories.

BMI < 18.5kgm²

Weeks	Min weight gain	Max weight gain		Min gain - 2kg cut point	Max gain + 2kg cut point		Min gain - 5kg cut point	Max gain + 5kg cut point
13	2	0.5		0	2.5		-3	5.5
14	2.39	1.15		0.39	3.15		-2.61	6.15
15	2.78	1.8		0.78	3.8		-2.22	6.8
16	3.17	2.45		1.17	4.45		-1.83	7.45
17	3.56	3.1		1.56	5.1		-1.44	8.1
18	3.95	3.75		1.95	5.75		-1.05	8.75
19	4.34	4.4		2.34	6.4		-0.66	9.4
20	4.73	5.05		2.73	7.05		-0.27	10.05
21	5.12	5.7		3.12	7.7		0.12	10.7
22	5.51	6.35		3.51	8.35		0.51	11.35
23	5.9	7		3.9	9		0.9	12
24	6.29	7.65		4.29	9.65		1.29	12.65
25	6.68	8.3		4.68	10.3		1.68	13.3
26	7.07	8.95		5.07	10.95		2.07	13.95
27	7.46	9.6		5.46	11.6		2.46	14.6
28	7.85	10.25		5.85	12.25		2.85	15.25
29	8.24	10.9		6.24	12.9		3.24	15.9
30	8.63	11.55		6.63	13.55		3.63	16.55
31	9.02	12.2		7.02	14.2		4.02	17.2
32	9.41	12.85		7.41	14.85		4.41	17.85
33	9.8	13.5		7.8	15.5		4.8	18.5
34	10.19	14.15		8.19	16.15		5.19	19.15

35	10.58	14.8		8.58	16.8		5.58	19.8
36	10.97	15.45		8.97	17.45		5.97	20.45
37	11.36	16.1		9.36	18.1		6.36	21.1
38	11.75	16.75		9.75	18.75		6.75	21.75
39	12.14	17.4		10.14	19.4		7.14	22.4
40	12.53	18.05		10.53	20.05		7.53	23.05

**Table create in excel I:\PregnancyAndReproduction\ShannaFealy\ExcessGWG_CRE20111\Data\Excel*

BMI 18.5 kgm² - <25kgm²

Weeks	Min weight gain	Max weight gain		Min gain - 2kg cut point	Max gain + 2kg cut point		Min gain - 5kg cut point	Max gain + 5kg cut point
13	2	0.5		0	2.5		-3	5.5
14	2.35	1.075		0.35	3.075		-2.65	6.075
15	2.7	1.65		0.7	3.65		-2.3	6.65
16	3.05	2.225		1.05	4.225		-1.95	7.225
17	3.4	2.8		1.4	4.8		-1.6	7.8
18	3.75	3.375		1.75	5.375		-1.25	8.375
19	4.1	3.95		2.1	5.95		-0.9	8.95
20	4.45	4.525		2.45	6.525		-0.55	9.525
21	4.8	5.1		2.8	7.1		-0.2	10.1
22	5.15	5.675		3.15	7.675		0.15	10.675
23	5.5	6.25		3.5	8.25		0.5	11.25
24	5.85	6.825		3.85	8.825		0.85	11.825
25	6.2	7.4		4.2	9.4		1.2	12.4
26	6.55	7.975		4.55	9.975		1.55	12.975
27	6.9	8.55		4.9	10.55		1.9	13.55
28	7.25	9.125		5.25	11.125		2.25	14.125
29	7.6	9.7		5.6	11.7		2.6	14.7

30	7.95	10.275		5.95	12.275		2.95	15.275
31	8.3	10.85		6.3	12.85		3.3	15.85
32	8.65	11.425		6.65	13.425		3.65	16.425
33	9	12		7	14		4	17
34	9.35	12.575		7.35	14.575		4.35	17.575
35	9.7	13.15		7.7	15.15		4.7	18.15
36	10.05	13.725		8.05	15.725		5.05	18.725
37	10.4	14.3		8.4	16.3		5.4	19.3
38	10.75	14.875		8.75	16.875		5.75	19.875
39	11.1	15.45		9.1	17.45		6.1	20.45
40	11.45	16.025		9.45	18.025		6.45	21.025

**Table create in excel I:\PregnancyAndReproduction\ShannaFealy\ExcessGWG_CRE20111\Data\Excel*

BMI 25kgm^2 - <30 kgm^2

Weeks	Min weight gain	Max weight gain		Min gain - 2kg cut point	Max gain + 2kg cut point		Min gain - 5kg cut point	Max gain + 5kg cut point
13	2	0.5		0	2.5		-3	5.5
14	2.19	0.907		0.19	2.907		-2.81	5.907
15	2.38	1.314		0.38	3.314		-2.62	6.314
16	2.57	1.721		0.57	3.721		-2.43	6.721
17	2.76	2.128		0.76	4.128		-2.24	7.128
18	2.95	2.535		0.95	4.535		-2.05	7.535
19	3.14	2.942		1.14	4.942		-1.86	7.942
20	3.33	3.349		1.33	5.349		-1.67	8.349
21	3.52	3.756		1.52	5.756		-1.48	8.756
22	3.71	4.163		1.71	6.163		-1.29	9.163
23	3.9	4.57		1.9	6.57		-1.1	9.57

24	4.09	4.977		2.09	6.977		-0.91	9.977
25	4.28	5.384		2.28	7.384		-0.72	10.384
26	4.47	5.791		2.47	7.791		-0.53	10.791
27	4.66	6.198		2.66	8.198		-0.34	11.198
28	4.85	6.605		2.85	8.605		-0.15	11.605
29	5.04	7.012		3.04	9.012		0.04	12.012
30	5.23	7.419		3.23	9.419		0.23	12.419
31	5.42	7.826		3.42	9.826		0.42	12.826
32	5.61	8.233		3.61	10.233		0.61	13.233
33	5.8	8.64		3.8	10.64		0.8	13.64
34	5.99	9.047		3.99	11.047		0.99	14.047
35	6.18	9.454		4.18	11.454		1.18	14.454
36	6.37	9.861		4.37	11.861		1.37	14.861
37	6.56	10.268		4.56	12.268		1.56	15.268
38	6.75	10.675		4.75	12.675		1.75	15.675
39	6.94	11.082		4.94	13.082		1.94	16.082
40	7.13	11.489		5.13	13.489		2.13	16.489

Table create in excel I:\PregnancyAndReproduction\ShannaFealy\ExcessGWG_CRE20111\Data\Excel

BMI ≥ 30kgm²

**Weeks**	**Min weight gain**	**Max weight gain**		**Min gain - 2kg cut point**	**Max gain + 2kg cut point**		**Min gain - 5kg cut point**	**Max gain + 5kg cut point**
13	2	0.5		0	2.5		-3	5.5
14	2.11	0.815		0.11	2.815		-2.89	5.815
15	2.22	1.13		0.22	3.13		-2.78	6.13
16	2.33	1.445		0.33	3.445		-2.67	6.445
17	2.44	1.76		0.44	3.76		-2.56	6.76
18	2.55	2.075		0.55	4.075		-2.45	7.075

19	2.66	2.39		0.66	4.39		-2.34	7.39
20	2.77	2.705		0.77	4.705		-2.23	7.705
21	2.88	3.02		0.88	5.02		-2.12	8.02
22	2.99	3.335		0.99	5.335		-2.01	8.335
23	3.1	3.65		1.1	5.65		-1.9	8.65
24	3.21	3.965		1.21	5.965		-1.79	8.965
25	3.32	4.28		1.32	6.28		-1.68	9.28
26	3.43	4.595		1.43	6.595		-1.57	9.595
27	3.54	4.91		1.54	6.91		-1.46	9.91
28	3.65	5.225		1.65	7.225		-1.35	10.225
29	3.76	5.54		1.76	7.54		-1.24	10.54
30	3.87	5.855		1.87	7.855		-1.13	10.855
31	3.98	6.17		1.98	8.17		-1.02	11.17
32	4.09	6.485		2.09	8.485		-0.91	11.485
33	4.2	6.8		2.2	8.8		-0.8	11.8
34	4.31	7.115		2.31	9.115		-0.69	12.115
35	4.42	7.43		2.42	9.43		-0.58	12.43
36	4.53	7.745		2.53	9.745		-0.47	12.745
37	4.64	8.06		2.64	10.06		-0.36	13.06
38	4.75	8.375		2.75	10.375		-0.25	13.375
39	4.86	8.69		2.86	10.69		-0.14	13.69
40	4.97	9.005		2.97	11.005		-0.03	14.005

**Table create in excel I:\PregnancyAndReproduction\ShannaFealy\ExcessGWG_CRE20111\Data\Excel*

Supplementary File S2: Cross tabulations describing the distribution of weight gain at visits 1, 2, 3 versus weight gain at visit 4.

	Time point/cut-point		*Normal GWG (N(%))*	*Excess/Inadequate GWG (N(%))*
2kg cut point	Visit 1 Refer	No referral	38 (59%)	16 (24%)
	Visit 1 Refer	Refer	26 (41%)	51 (76%)
	Visit 2 Refer	No referral	46 (72%)	13 (19%)
	Visit 2 Refer	Refer	18 (28%)	54 (81%)
	Visit 3 Refer	No referral	54 (84%)	6 (9.0%)
	Visit 3 Refer	Refer	10 (16%)	61 (91%)
	Consecutive +/- 2kg at Visit 1 and 2	No referral	48 (75%)	18 (27%)
	Consecutive +/- 2kg at Visit 1 and 2	Refer	16 (25%)	49 (73%)
	Consecutive +/- 2kg at Visit 2 and 3	No referral	57 (89%)	15 (22%)
	Consecutive +/- 2kg at Visit 2 and 3	Refer	7 (11%)	52 (78%)
5 kg cut point	Visit 1 Refer	No referral	86 (91%)	22 (59%)
	Visit 1 Refer	Refer	8 (8.5%)	15 (41%)
	Visit 2 Refer	No referral	88 (94%)	13 (35%)
	Visit 2 Refer	Refer	6 (6.4%)	24 (65%)
	Visit 3 Refer	No referral	91 (97%)	5 (14%)
	Visit 3 Refer	Refer	3 (3.2%)	32 (86%)
